# Supplementary figures and images for: Pseudomonas aeruginosa induces p38MAP kinase-dependent IL-6 and CXCL8 release from bronchial epithelial cells via a Syk kinase pathway
Source: PLoS One. 2021 Feb 1;16(2):e0246050. doi: 10.1371/journal.pone.0246050 (PMC7850485; doi:10.1371/journal.pone.0246050)

Figure 4 raw images

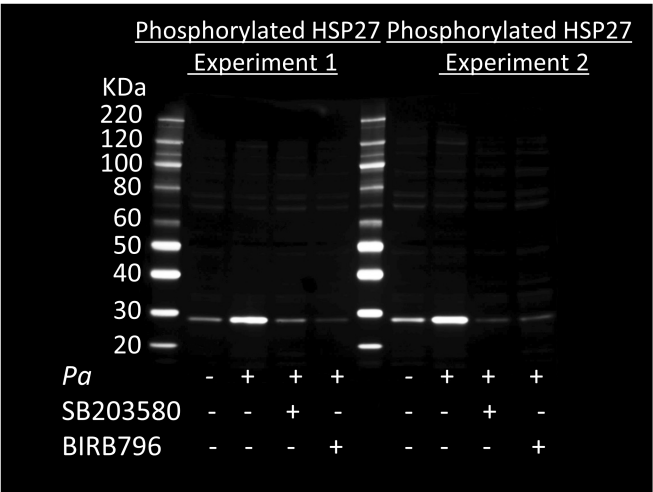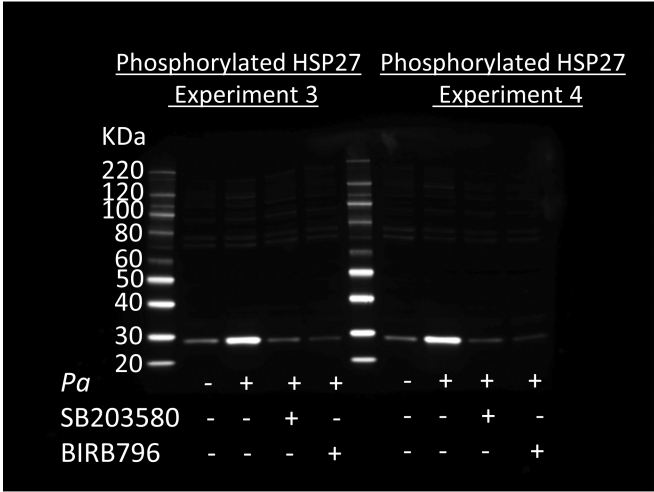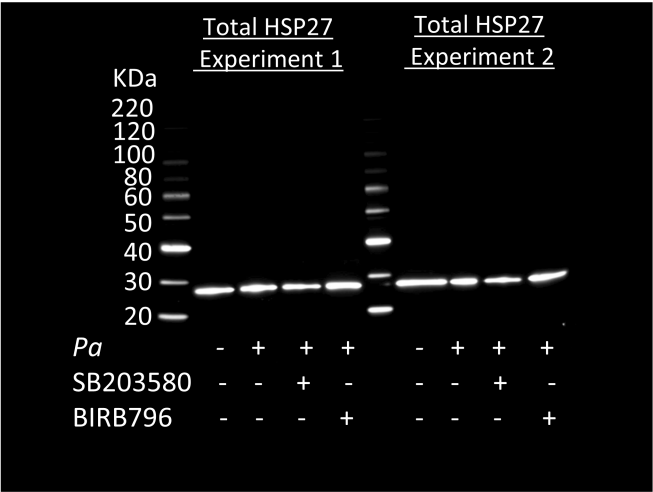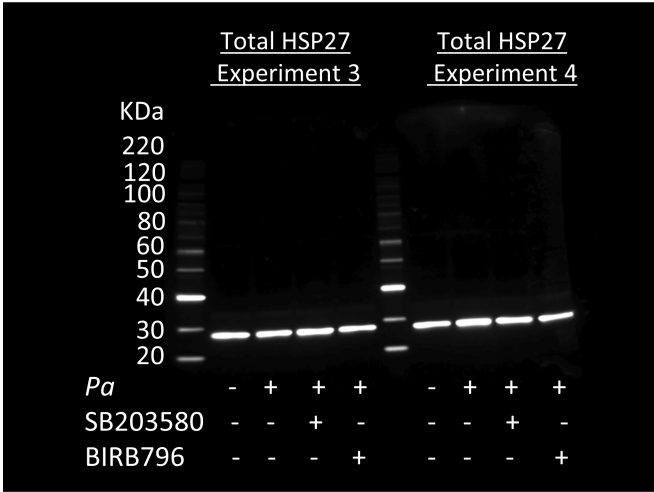

Figure 5A raw images

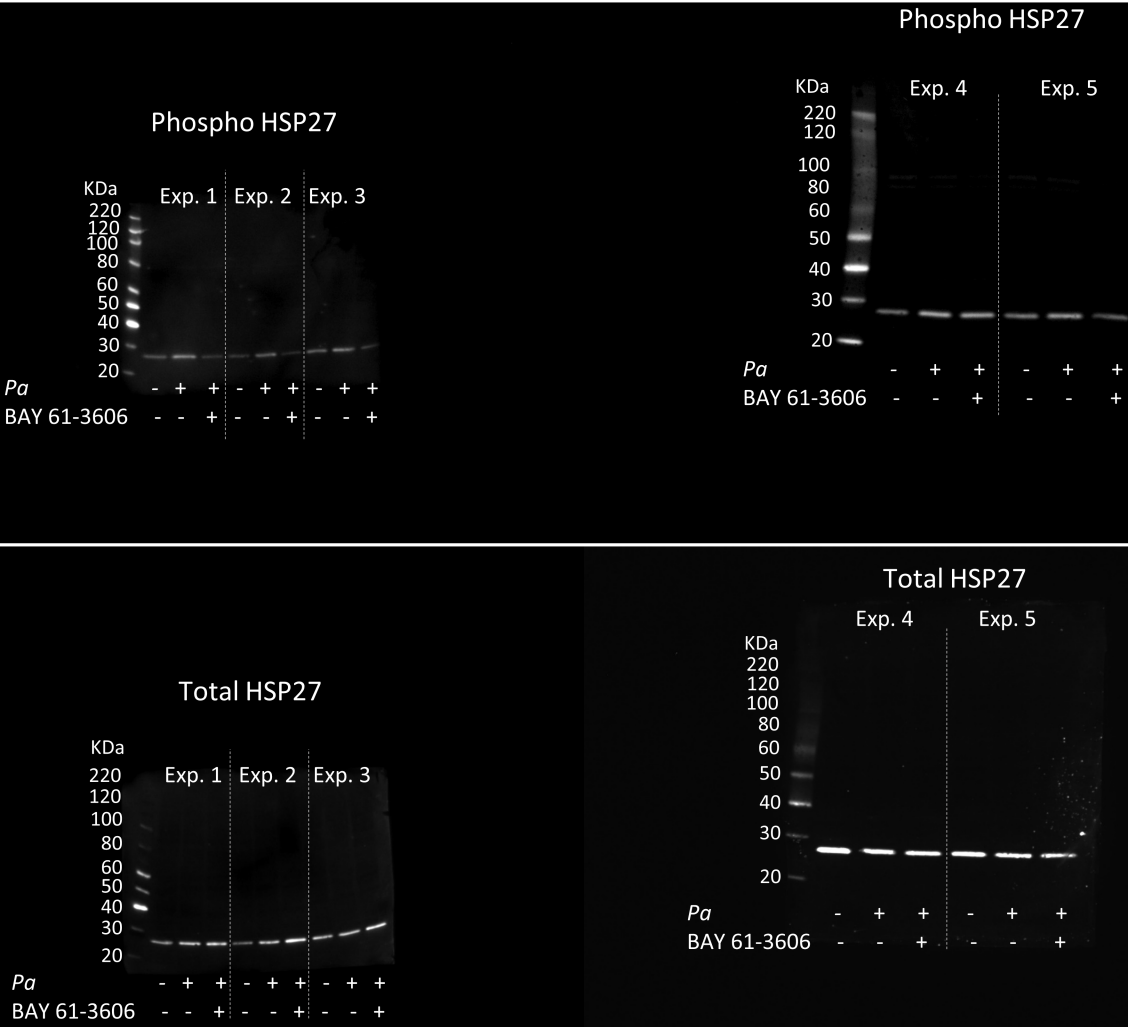

Figure 5B raw images

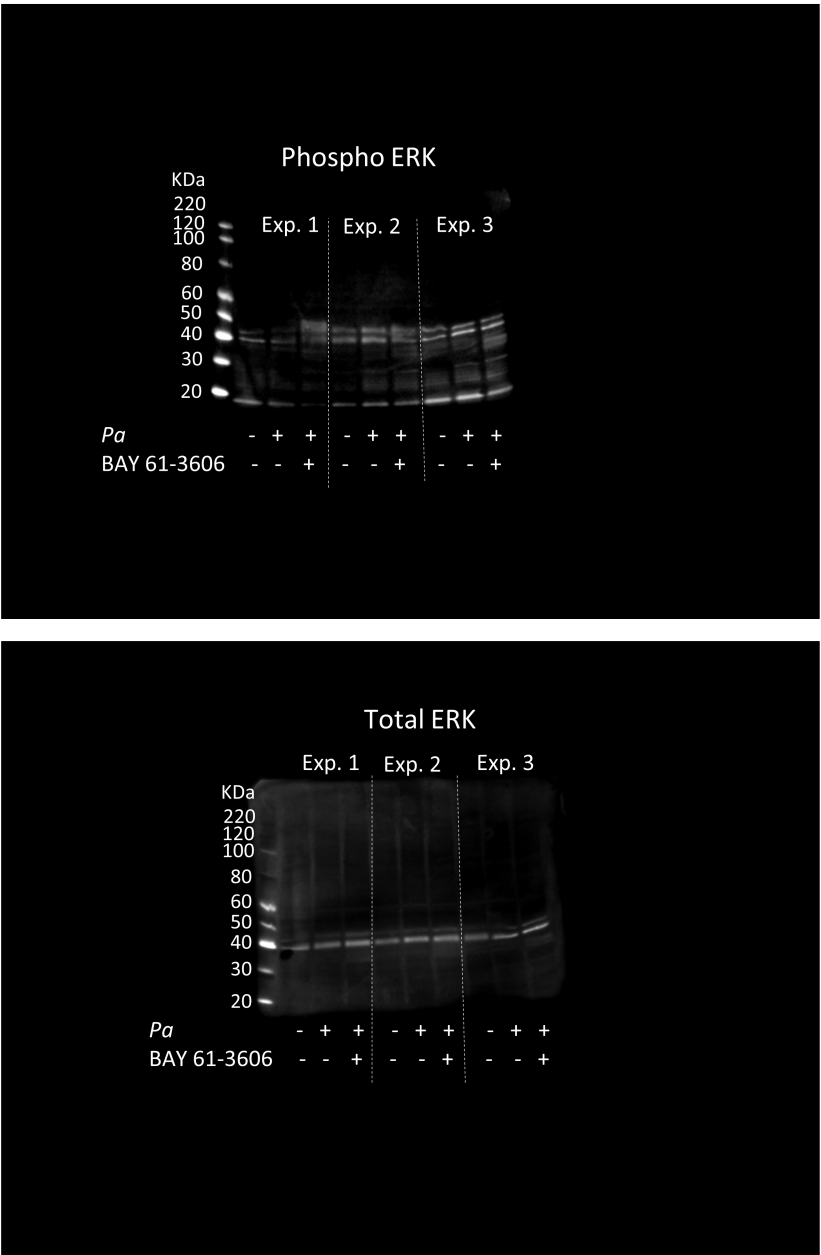

Figure 5C raw images

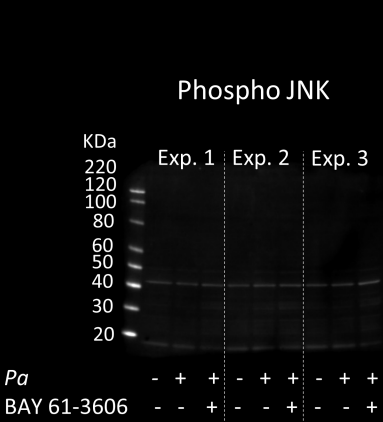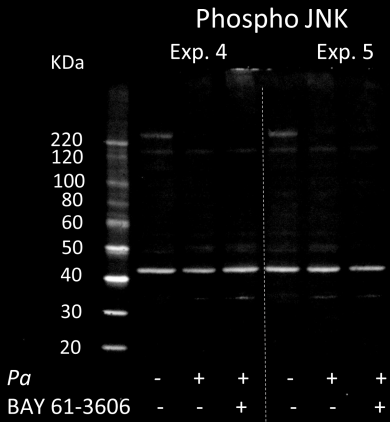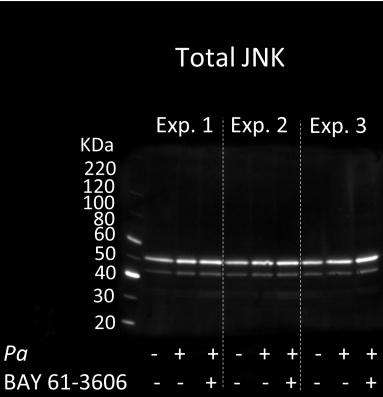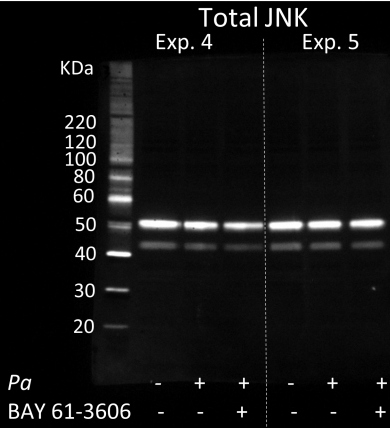

Supplement: S1 File — (PDF) [file pone.0246050.s001.pdf]
