# Supplementary material for: Pseudomonas aeruginosa induces p38MAP kinase-dependent IL-6 and CXCL8 release from bronchial epithelial cells via a Syk kinase pathway
Source: PLoS One. 2021 Feb 1;16(2):e0246050. doi: 10.1371/journal.pone.0246050 (PMC7850485; doi:10.1371/journal.pone.0246050)
Supplement: Attachment — Submitted filename: Coates et al. review.docx [file pone.0246050.s002.docx]

In this manuscript by Coates et al., the authors conclude that Pseudomonas aeruginosa (Pa) infection triggers IL-6/CXCL8 release in bronchial epithelial cells through Syk and p38 MAPK signaling pathways. Overall, the data support this conclusion, although there are several remaining issues with the manuscript.

General Concerns:

1. The figures are very low resolution and almost impossible to read. It makes it very difficult to closely examine or interpret the data within this manuscript. These figures need to be vector graphic files to rectify the pixelation and blurriness.
2. It’s hard to tell given the poor resolution, but indicators of statistical significance appear on some but not all of the graphs. Including this on all graphs would increase clarity.
3. I do not see the value of Table 2 or 3. What exactly do they provide that the graphs (obviously once the resolution is improved) do not? An additional graph showing % inhibition could be included instead of Table 3 if truly desired. The tables also lack legends clarifying which experiments they are tied to.
4. Some small typos/errors remain in the manuscript, the text should be carefully edited prior to publication

Specific Concerns:

1. The intro refers to the common CF mutation Phe508del but does not reference the specific gene (*CFTR*).
2. I cannot read the axis labels for Fig. 2, but regardless the concentrations of the inhibitors should be stated in the figure legends. It appears to say 1nM for BAY 61-3606? If so, that is equal to ~0.5ng/ul, which is dramatically lower than the reported IC_50_ in Fig. 3C (~350ng/ul). This apparent discrepancy is not addressed.
3. The authors state “*However, at a concentration of BIRB796 that did not give complete inhibition of IL-6 or CXCL8 there was complete inhibition of p38MAPK α and β activity (p<0.01, Figs 4A and B).*” This statement is unclear because 1ug/ml was used for both compounds in Fig. 4, yet this concentration was highest dose used in Fig. 3 and showed almost complete inhibition of IL-6 and CXCL8 release.
4. The authors also state “*Activity of SB203580 suggests that p38MAPK α and β have an important role in both Pa-induced IL-6 and CXCL8 release and the difference between SB203580 and BIRB796 suggests that the δ and γ isoforms are also involved*.” This statement is not supported by the data in Fig. 4. All the data in Fig. 3 show is that BIRB796 is a more potent inhibitor of IL-6 and CXCL8 release. This could simply be because it’s a better inhibitor of p38α.
5. In order to determine the effect of BAY 61-3606 on p38MAPK activity, p38 activation should be analyzed with a phospho-p38 antibody. It would also be informative to determine whether Pa results in Syk activation using a phospho-Syk antibody. Does p38 inhibition reciprocally affect Syk activation?
6. The protein ladder lanes in Fig 5 are not labeled.
7. The IC_50_s of RV1088 should be determined and compared with co-treatment with BIRB796/BAY 61-3606.
8. RV1088 and/or BIRB796/BAY 61-3606 should also be tested in CFTR (Phe508del) expressing bronchial epithelial cells to evaluate its therapeutic potential.
