# Supplementary material for: Pseudomonas aeruginosa induces p38MAP kinase-dependent IL-6 and CXCL8 release from bronchial epithelial cells via a Syk kinase pathway
Source: PLoS One. 2021 Feb 1;16(2):e0246050. doi: 10.1371/journal.pone.0246050 (PMC7850485; doi:10.1371/journal.pone.0246050)
Supplement: Attachment — Submitted filename: Response to Reviewers.docx [file pone.0246050.s004.docx]

Dear Dr Bratton,

RE: **Revision for PONE-D-20-23493R1** - *Pseudomonas aeruginosa* induces p38MAP kinase-dependent IL-6 and CXCL8 release from bronchial epithelial cells via a Syk kinase pathway

We would like to thank the Editor and Reviewer for their comments, which we have carefully considered in preparing this revised manuscript.

We have addressed the reviewers’ comments point-by-point below. All authors concur with the submission and the material submitted for publication has not been previously reported and is not under consideration for publication elsewhere. Please note, line numbers stated in this letter refer to the file named “Revised Manuscript with Track Changes.”

Reviewer 1

[Comment 1]

The western blot of Fig. 4A has lines between each lane which makes it appear as though it was stitched together from multiple different blots? That would not be suitable for interpretation or publication.

We apologise; the lines were added to make the distinction between lanes clearer, but understand that this could cause confusion. We have, therefore, removed these.

[Comment 2]

Relatedly, the authors chose to focus on my comment #8, which does add therapeutic relevance to the manuscript. However, in the text they do not address the discrepancies with previously published data. In the discussion the authors cite earlier published data stating that IL-6 release is higher in Phe508del CFTR-expressing cells compared to wild-type, but in their hands it was the opposite.

Thank you for this comment and we accept the discrepancy between the results observed in this study and those from previous studies that are included in the discussion. We have addressed this in lines 488-496 with the statement "The current study did not show greater Pa-induced IL-6 from epithelial cells expressing Phe508del CFTR, compared with WT. This agrees with previous research showing little difference between IL-6 induction from WT and CF primary epithelial cells and cell lines by multiple stimulants (65,66). This however, is counter to the higher Pa-induced IL-6 secretion by Phe508del CFTR expressing cells reported by Berube et al. (22). However, that study used different cell lines, with the CF and WT cells originally from different donors and which were, therefore, not paired. Also, Berube et al. used sterile culture filtrates rather than whole cells, the study, therefore, not being directly comparable to the live infection used in the current study.”

Yours sincerely,

Matthew Coates
